# A UAV Testbed for Diagnosing Hardware Vulnerabilities: Quantifying Sim-to-Real Discrepancies in PX4 Flight Logs

**DOI:** 10.3390/s26103188

**Published:** 2026-05-18

**Authors:** Kubra Kose, Jacob Wing, Nuri Alperen Kose, Carlos Guadarrama-Trejo, Ayden Sowers, Amar Rasheed

**Affiliations:** Department of Computer Science, Sam Houston State University, Huntsville, TX 77341, USA

**Keywords:** UAV testbed, sim-to-real gap, cyber–physical security, hardware vulnerabilities, PX4 autopilot, uORB telemetry, anomaly detection baseline, flight data analysis

## Abstract

This paper presents a comprehensive UAV testbed that establishes quantitative baselines for hardware vulnerability diagnosis and cyber–physical security validation by leveraging comparative flight logs from PX4-based Software-In-The-Loop (SITL) simulations and multiple real-world quadrotor missions. The testbed utilizes a unified data pipeline centered on the uORB message bus and ULog format, enabling the extraction of high-resolution telemetry, including raw Inertial Measurement Unit (IMU) data, state-estimation, and actuator control signals. Evaluated across varying environmental conditions, side-by-side time-series and statistical analyses reveal critical sim-to-real discrepancies in sensor fidelity, GPS interference, and onboard resource behavior that are often overlooked in virtual environments. Real-world data exposes hardware-induced noise, mechanical vibrations, and electromagnetic disturbances that significantly impact flight stability and system reliability. By mathematically quantifying these discrepancies (e.g., via variance and probability distribution shifts), the proposed testbed establishes a rigorous baseline for distinguishing natural physical variability from anomalous or adversarial behavior. Ultimately, this work provides a foundational framework for developing robust anomaly detection models and validating the cyber–physical security of autonomous UAV systems in safety-critical environments.

## 1. Introduction

Unmanned Aerial Vehicles (UAVs) have emerged as a platform that is agile and sensor-rich and supports data-driven operations across a wide range of settings, from dense urban areas to remote disaster zones. In surveillance, mapping, infrastructure inspection, and emergency response, where precise control and dependable sensor feedback are critical for safe, autonomous flight, their versatility has greatly aided progress [1]. In order to enable this autonomy, open-source autopilot stacks, such as PX4, ArduPilot, and Paparazzi, provide real-time flight control, mission planning, and failsafe logic on low-cost embedded hardware. Software-In-The-Loop (SITL) and Hardware-In-The-Loop (HITL) simulations are essential for rapid prototyping of these autopilots due to their tight integration with companion-computer middleware (e.g., ROS, MAVSDK) [2,3].

Among the many robotic simulators available, Gazebo, Webots, and CoppeliaSim are the most popular, as they strike a balance between physical realism, sensor fidelity, and ROS integration [4]. Nevertheless, even state-of-the-art simulators struggle to emulate real-world characteristics such as unmodeled sensor noise, hardware-induced delays, wind gusts, and ground-effect aerodynamics.

While the sim-to-real gap in aerodynamics is well-documented, its impact on cybersecurity and hardware anomaly detection remains a critical, unsolved challenge. To bridge this simulation-to-reality divide, recent advancements have increasingly explored Physics-Informed Machine Learning (PIML) techniques to integrate physical constraints and real-world dynamics directly into predictive models and system validation [5,6]. However, the success of these advanced sim-to-real methodologies in a security context fundamentally depends on accurately defining the underlying hardware noise floor. As recent literature emphasizes, security mechanisms, such as Artificial Intelligence-based Intrusion Detection Systems (IDS), that are trained in idealized simulations suffer from severe covariate shifts. This causes their real-world performance to drop catastrophically (e.g., down to a 21% F1-score) when exposed to actual physical telemetry noise [1]. Furthermore, existing datasets for UAV fault and anomaly detection are incredibly scarce, hindering the development of resilient security models [7]. Therefore, when control, perception, and security algorithms are transferred from virtual testbeds to physical aircraft, these mismatches produce not only unsafe aerodynamic behaviors but also severe, unpredictable cybersecurity vulnerabilities [8,9].

Consequently, the foundation for any reliable security validation must be strictly defined by the threshold of natural physical variance. In a False Data Injection (FDI) or sensor spoofing attack, a sophisticated adversary will attempt to manipulate telemetry (e.g., inducing GPS drift or Inertial Measurement Unit (IMU) bias) while remaining below the detection threshold of onboard monitors. If an IDS is trained exclusively on idealized simulation data (where variance is near zero), it will trigger constant false positives when exposed to benign environmental noise. Conversely, if the IDS detection threshold is arbitrarily raised to account for the real world without mathematical justification, attackers can exploit this widened blind spot to hide malicious hardware attacks. By formally quantifying the environmental discrepancy (via Variance, RMSE, and JSD), this work establishes the exact quantitative baseline required for security validation, defining the boundaries within which an IDS must successfully distinguish between natural aerodynamic disturbances and active hardware exploitation.

By comparing PX4-based SITL simulations directly with matched quadrotor flights in the real world, we rigorously quantify this physical discrepancy. Telemetry and sensor data are captured and visualized using similar mission profiles and unified logging pipelines, including velocity, thrust, motor outputs, inertial and magnetic readings, GPS interference, angular rates, and onboard resources. The comparison of these metrics allows researchers to characterize simulator fidelity, diagnose control or estimation discrepancies, and iteratively refine both simulation models and real-flight controllers.

The primary novelty of this research lies in isolating and mathematically quantifying the microsecond-level physical hardware noise floor to establish a strictly uncalibrated baseline for cyber–physical security validation. To address the critical gap between macroscopic aerodynamic simulation and microsecond-level physical reality, our proposed testbed makes three novel contributions:

- We utilize the high-frequency logging capabilities native to PX4 to formally isolate and quantify the un-resampled “noise floor” of physical hardware (e.g., raw IMU and actuator telemetry). This advances beyond prior observational studies by establishing the strict mathematical boundaries required for cyber–physical security validation.
- We provide a rigorous statistical methodology, evaluating non-parametric significance and probability distribution shifts, to explicitly quantify the exact magnitude of the sim-to-real discrepancy.
- We establish this mathematically quantified discrepancy as the mandatory baseline (Quantitative Threat Model) for evaluating Artificial Intelligence-based IDS, ensuring future security algorithms are resilient to real-world physical noise rather than idealized virtual data.

In the following sections, Section 2 reviews related work on UAV simulation fidelity, sensor modeling, and sim-to-real transfer challenges. Section 3 outlines the UAV testbed architecture, including onboard hardware and flight control components. Section 4 explains the simulation environment configuration using PX4 v1.15.2 and Gazebo v1.16.0. Section 5 describes the dataset sources and flight metrics collected. Section 6 compares the real and simulated UAV behavior across multiple dimensions. Section 7 presents a discussion of the results and testbed limitations. Section 8 provides the concluding remarks regarding the sim-to-real gap and its implications for cyber–physical security. Finally, Section 9 outlines planned future directions, including large-scale fault-injection datasets and the development of anomaly detection models.

## 2. Literature Review

As real-world testing is limited by safety, cost, or logistical constraints, UAV simulation platforms have become increasingly important for developing and validating autonomous flight algorithms. Several frameworks have emerged to bridge the gap between simulation and field deployment, but differences between simulated and actual UAV behavior remain.

A significant portion of recent UAV simulation research has heavily prioritized visual navigation, SLAM, and obstacle avoidance. For instance, Chen et al. [10] introduced an end-to-end ROS-Gazebo-PX4 platform for visual SLAM, while Nguyen and Nguyen [11] proposed a vision-based SITL setup for landing pad detection. Similarly, platforms like XTDrone [12] and environments utilizing Unreal Engine and AirSim [13,14] have advanced multi-UAV object detection, stereo depth camera noise modeling, and photorealistic 3D reconstruction. However, while these platforms demonstrate strong support for vision-based algorithmic testing, their contributions are primarily macroscopic and photorealistic. They consistently lack detailed benchmarking against real-world UAV hardware dynamics and fail to address the microsecond-level sensor noise and hardware fidelity gaps central to our testbed.

To capture environmental variability, custom plugins have been developed by researchers such as Imamov et al. [8] to model conditions like wind within the Gazebo-PX4 simulation, emphasizing the importance of parameter tuning to approximate physical dynamics. Beyond environmental physics, system-level test generators have been introduced to automate and scale UAV testing. For example, Khatiri et al. [15] proposed Aerialist, a simulation-based tool that executes flight log analysis in Kubernetes environments, highlighting the growing necessity of replicating real-flight logs for simulation realism.

Despite these advancements, broad surveys of simulation platforms, such as the comprehensive review by Gill et al. [2], frequently identify a persistent lack of integrated testbeds that span both network and physical simulation. To systematically bridge these isolated environments, recent methodologies include the robust, four-stage testing pipeline presented by Jiang et al. [3], which progresses sequentially through SITL, HITL, controlled indoor trials, and full in-field validation. Their practical integration of ROS and PX4 with simulators like AirSim provides valuable insight into physical failure scenarios and iterative controller improvements.

While early simulation platforms emphasized visual fidelity for navigation, contemporary research has urgently shifted towards cyber–physical security and hardware-level validation. For instance, Pant et al. [16] developed MIXED-SENSE, a mixed-reality sensor emulation framework using Gazebo to evaluate UAV resilience against FDI and GNSS spoofing attacks. However, their approach relies on high-end external motion capture systems rather than quantifying the intrinsic physical noise floor of the embedded hardware itself.

Addressing the sim-to-real gap directly, Coursey et al. [17] quantified the transferability of a UAV disturbance rejection controller by evaluating the Jensen–Shannon Divergence (JSD) between simulated and real-world measurement distributions. Their methodology underscores the necessity of distribution-level metrics, yet our proposed testbed extends this statistical rigor specifically to high-frequency uORB telemetry for security validation.

In the domain of cyber-attack detection, Wang et al. [18] proposed QUADFormer, demonstrating that traditional statistical detectors fail under the highly non-linear dynamics of physical quadrotors. By utilizing Extended Kalman Filter (EKF) residues, their architecture detects sparse FDI attacks. Similarly, Tan et al. [19] introduced RADD, combining rule-mining with unsupervised learning to detect runtime anomalies without overfitting to a single simulated environment. Furthermore, prioritizing hardware vulnerabilities, Almeida et al. [20] demonstrated the critical threat of Hardware Trojans (HTs) on UAV flight controllers, utilizing side-channel impedance analysis to detect malicious Pulse Width Modulation (PWM) signal inversions.

Complementing this focus on embedded security, recent work by Rasheed et al. [21] introduced an IoT-based system architecture for in-field anomaly detection in UAVs using a distributed LSTM model trained via energy-aware edge devices. While their focus lies in cyber threat mitigation and adaptive IDS design, their system aligns with our findings by emphasizing the importance of deploying real-time, lightweight testing and validation pipelines that function beyond simulated environments.

Together, these studies highlight a persistent gap in the literature: while macroscopic aerodynamic and vision-based simulations have matured significantly, microsecond-level hardware telemetry validation remains largely overlooked. Existing testbeds frequently assume idealized sensor fidelity, which inadvertently conceals the physical noise floor required to evaluate cyber–physical vulnerabilities. Our proposed testbed complements these existing platforms by offering detailed, quantitative comparisons of IMU variance, Radio Frequency (RF) interference, and actuator control behavior. By mathematically bounding these physical discrepancies, this work provides the exact baselines required to not only calibrate flight simulations, but to successfully train and transfer resilient cyber–physical anomaly detectors to real-world deployments.

## 3. Testbed Architecture Overview

This section describes the physical and software components of the proposed UAV testbed, detailing the onboard hardware, PX4-based flight stack, and ground-control interface that collectively enable the sim-to-real experiments presented later in the paper. It is critical to distinguish that this proposed architecture constitutes a holistic testbed, rather than a mere data-processing workflow. The testbed is defined by the strict, matched integration of the physical NXP quadrotor hardware, the precisely mirrored SITL Gazebo environment utilizing identical EKF2 state-estimation, and the automated uORB telemetry extraction pipeline. Together, these components form a robust, repeatable experimental platform capable of executing and quantifying parallel cyber–physical missions across varying environmental conditions and flight profiles.

### 3.1. Hardware

Our UAV testbed is built around the NXP RDDRONE-FMUK66 Flight Management Unit (FMU), which incorporates a Kinetis K66 MCU (NXP Semiconductors, Eindhoven, The Netherlands) based on the Arm Cortex-M4 architecture, operating at 180 MHz. The FMU features integrated sensors, including an accelerometer, a gyroscope, a barometer, and a magnetometer. It receives power through a Readytosky APM power module, which draws energy from a 3 S 11.1 V 5600 mAh LiPo battery (120 C) (Sankaku, Shenzhen, China).

A Holybro M8N GPS module is connected to the FMU via a JST-GH 10-pin connector (J.S.T. Mfg. Co., Ltd., Osaka, Japan) and mounted on the airframe. For radio control, the drone is equipped with a FlySky FS-iA6B receiver (Shenzhen Flysky Technology Co., Ltd., Shenzhen, China), operating at 2.4 GHz across 6 channels, with rear-facing antennas for signal redundancy. During the comparative evaluation, this RF link is utilized strictly as a failsafe override, ensuring that the evaluated telemetry reflects pure autonomous trajectory tracking rather than human pilot jitter.

**Figure 1:**
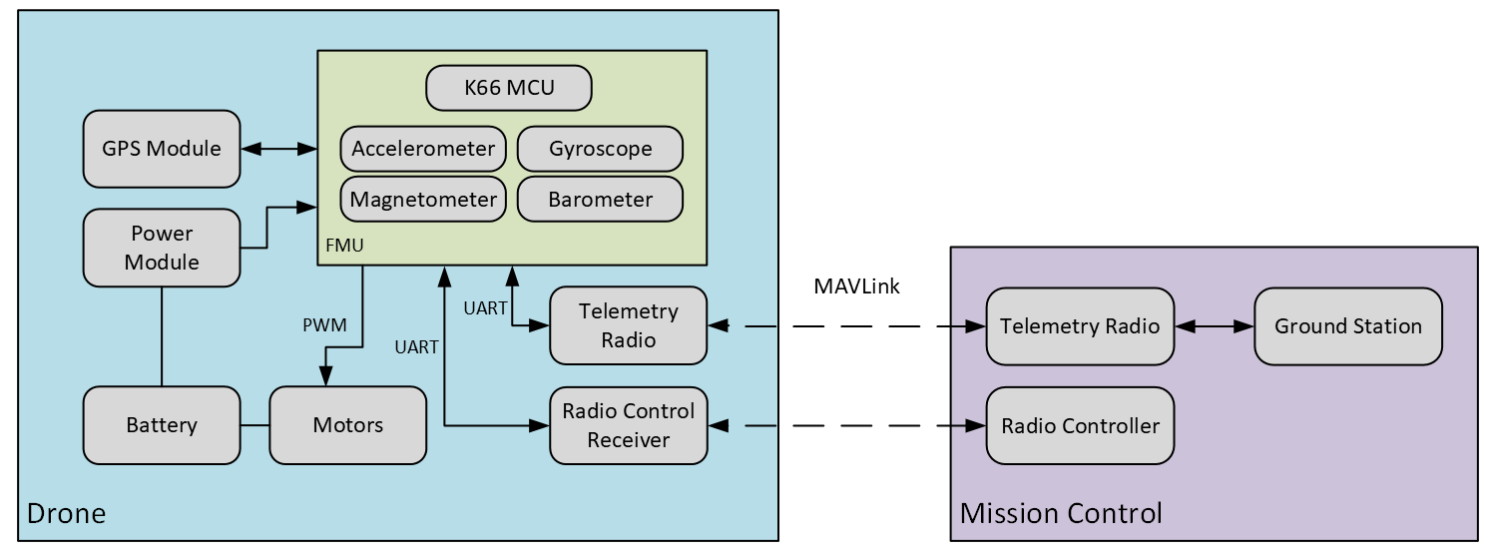
Drone hardware architecture with FMU, sensors, power, and communication modules. Solid arrows indicate wired onboard power and data connections (e.g., UART, PWM), while dashed arrows represent wireless communication links. Color-coded background panels distinguish the subsystems: the blue panel indicates the overall physical onboard drone components, the purple panel indicates the external Mission Control ground station, and the inner green panel specifically highlights the FMU and its tightly integrated internal components (MCU and sensors).

Telemetry communication is achieved using a pair of Holybro 915 MHz (Holybro, Shenzhen, China) telemetry radios, one onboard and one on the ground station. The UAV utilizes four FlyCat 5010 750 KV brushless motors, (FlyCat, Shenzhen, China), each equipped with 12-inch Ancalos LJI 1245 propellers (LJI, Shenzhen, China), arranged in a “props-in” configuration. Figure 1 illustrates the hardware architecture of the UAV system, showing the integration between the FMU, power supply, sensors, telemetry modules, and the ground station.

**Figure 2:**
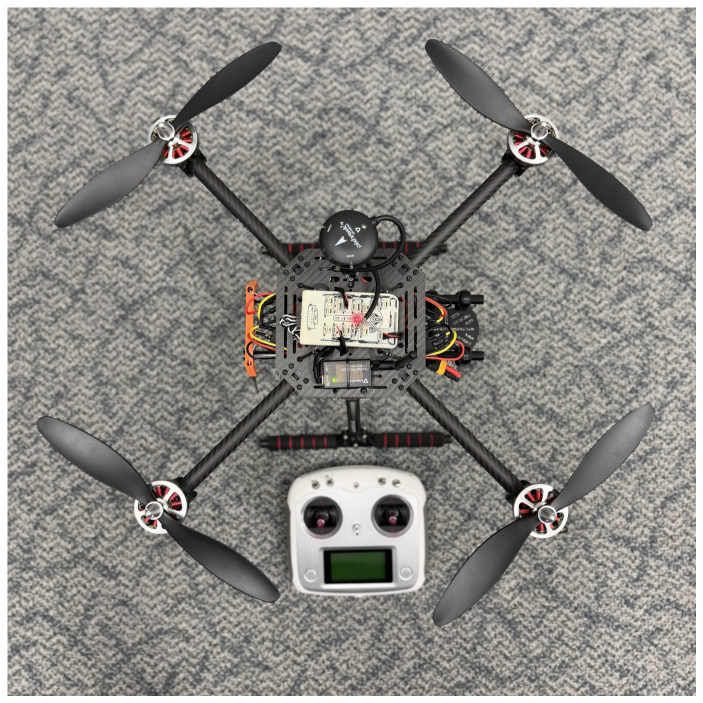
Top–down view of the quadcopter platform used in this study.

The quadcopter platform used in this study includes brushless motors with propellers, electronic speed controllers (ESCs), a Li-Po battery mounted below the top plate, a GPS module with integrated compass, a telemetry radio with antenna, an RC receiver, and a PX4-based flight controller located at the center of the frame. Figure 2 shows the complete system from a top–down view.

To ensure baseline measurement accuracy and mitigate the impact of sensor miscalibration, a comprehensive sensor calibration suite (covering the accelerometer, gyroscope, magnetometer, and horizon level) is strictly executed via QGroundControl prior to field deployments. By establishing this normalized baseline, the testbed ensures that the high-frequency variance and divergence observed during subsequent data analysis stem purely from dynamic physical environmental factors (e.g., aerodynamic drag and mechanical vibration) rather than static hardware offsets.

### 3.2. Flight Stack

The onboard software stack is based on the PX4 Autopilot firmware, which runs on the NuttX Real-Time Operating System (RTOS) v12.0.0. The architecture is composed of two main layers: a middleware layer for low-level communication and a high-level flight control stack.

#### 3.2.1. Middleware Layer

This layer includes device drivers for sensor interfacing, MAVLink communication modules for external data exchange, and the uORB message bus, which enables asynchronous inter-process communication. It is responsible for acquiring sensor data, routing internal messages, and logging flight telemetry. From a cybersecurity perspective, the uORB bus represents the primary internal attack surface; if an adversary executes a FDI attack on a peripheral sensor, the malicious payloads propagate through uORB to the flight controller. Consequently, tapping directly into uORB for high-frequency ULog extraction provides the exact data layer required to monitor cyber–physical exploits.

#### 3.2.2. Flight Stack Layer

The flight stack comprises the system’s decision-making components. It includes state machines to manage mission phases, control algorithms for flight stabilization, and navigation algorithms to execute autonomous waypoints. Commands from the Ground Control Station (GCS) are received via MAVLink, processed by the middleware, and forwarded to the control layer through the uORB bus. Real-time actuator signals are then generated, and flight data is concurrently logged on an onboard SD card.

**Figure 3:**
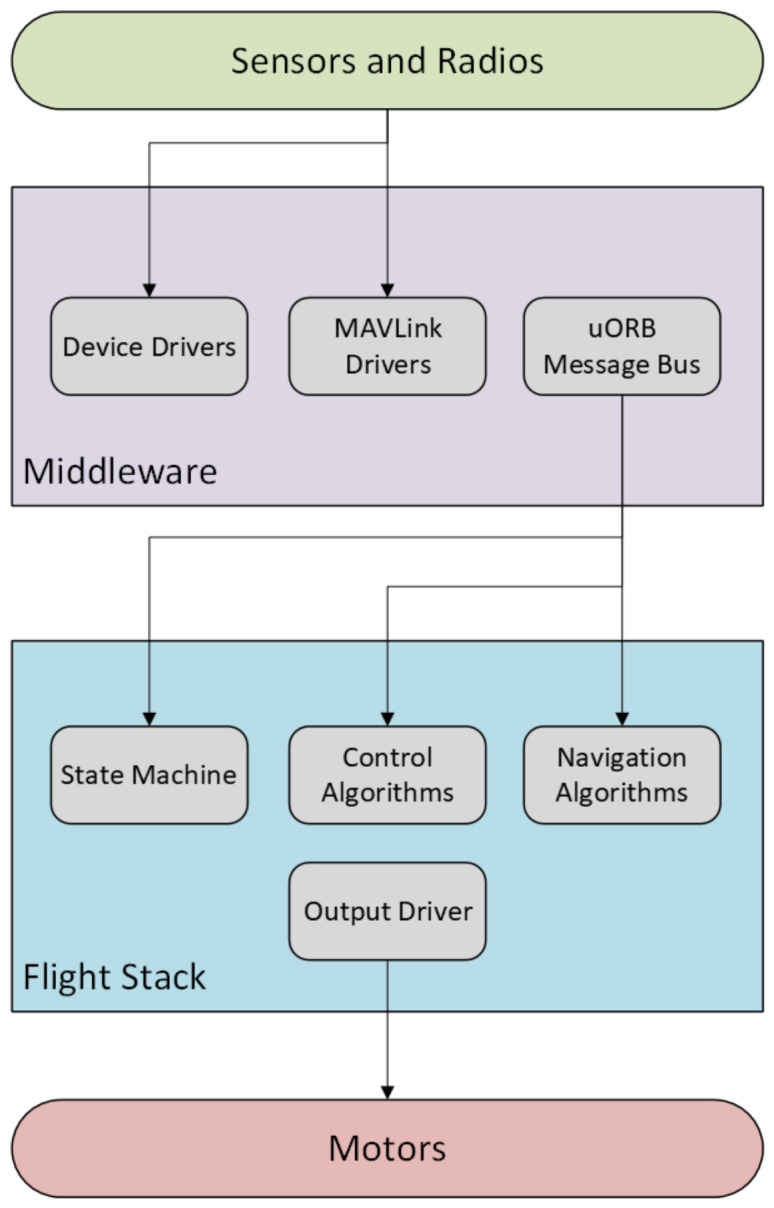
PX4 Flight Stack SoftwareArchitecture.

Figure 3 displays the PX4 software stack with middleware and control logic layers separated, as well as the flow of communication between the modules.

### 3.3. Ground-Control Station

We used QGroundControl (QGC) v4.4.0 as the ground station software, which interfaces with the drone via MAVLink over a 915 MHz telemetry radio. QGC allows mission planning, parameter tuning, and real-time visualization of telemetry data. It also enables logging of SITL-based simulated missions and real-world flight logs in PX4-compatible ULog format.

## 4. Simulation Environment Setup

This section outlines the complete SITL simulation environment used to mirror the drone’s real-world behavior. The simulation environment consists of the PX4 Autopilot, Gazebo simulator, QGroundControl, and ROS 2 nodes to collect and visualize telemetry data [22,23,24,25].

### 4.1. Software-in-the-Loop (SITL) Architecture

PX4 supports SITL simulation, which allows the full PX4 firmware to be executed on a host PC using Gazebo as the simulation environment. This eliminates the need for a physical flight controller and enables testing of control algorithms, mission planning, and sensor emulation without risk to hardware.

### 4.2. Gazebo Simulation Configuration

The SITL environment was configured utilizing the PX4 firmware executed alongside the Gazebo simulator. This specific architecture ensures that the simulated flight controller operates with the exact same codebase and state-estimation logic (EKF2) as the physical hardware. To bridge the PX4 middleware with external logging and control tools, ROS 2 was integrated using the Micro XRCE-DDS agent, enabling the real-time, high-frequency telemetry exchange required for our offline evaluation pipeline. Finally, the Gazebo simulation location was set to be the exact same as the real-world flight area to ensure magnetic and environmental consistency during the comparative analysis.

To establish an objective baseline for quantifying the sim-to-real gap, the simulation was deliberately executed using the default PX4_SITL Generic Quadrotor parameter set rather than performing a custom parameter tuning sweep. Modifying the simulation to artificially match physical flight would obscure the natural environmental discrepancies critical to our Threat Model. Consequently, the baseline Multicopter Position Control (MPC) parameters were fixed, utilizing standard proportional position gains (MPC_XY_P = 0.95, MPC_Z_P = 1.0) and velocity acceleration limits (MPC_XY_VEL_P_ACC = 1.8, MPC_Z_VEL_P_ACC = 4.0). Furthermore, the Extended Kalman Filter (EKF2) noise models were configured with idealized default thresholds, including gyroscopic noise (EKF2_GYR_NOISE = 0.015) and magnetic noise (EKF2_MAG_NOISE = 0.05 Gauss). By strictly freezing these parameters across the evaluation, we ensure that the quantified statistical divergence (e.g., Variance and JSD) purely reflects physical hardware dynamics and aerodynamic turbulence rather than algorithmic compensation. Additionally, no synthetic stochastic RF disturbance or GNSS spoofing plugins were loaded into the Gazebo environment. Consequently, the simulation provides a mathematically pure, zero-interference baseline, which is essential to identifying the true electromagnetic noise floor experienced by the physical hardware.

## 5. Data Collection and Feature Overview

This section presents a comprehensive overview of the metadata and telemetry features collected from both real-world and simulated drone operations. PX4’s ULog format was used to log all data and later parsed for comparative analysis. To ensure a strictly controlled quantitative baseline, both the simulated and physical UAV were commanded using the identical QGroundControl mission plan, guaranteeing that the target trajectory and autonomous control setpoints were perfectly matched prior to takeoff. The field validation missions for the real-world dataset were conducted at a Federal Recreational Flyer Fixed Site (FRIA ID: 2023-CBO-02209-FRIA) [26]: Sky High RC Park #3215 — Erwin Park, located at 3734 Co Rd 164, McKinney, TX 75071. This site, affiliated with the Academy of Model Aeronautics, is designated for recreational operations with a geographical center at 33.25° N, −96.66° W (McKinney, TX). Flight operations were conducted under the site’s ceiling limit of 700 feet AGL (Above Ground Level), ensuring compliance and providing a controlled operational environment for collecting real-life drone data.

### 5.1. Flight Metadata Summary

To capture the microsecond-level nuances of cyber–physical behavior, the primary visual distributions and time-series analyses in this study are derived from a single, detailed representative flight pair. However, to explicitly address inter-flight variability and ensure the testbed’s robustness, the core statistical variance evaluations were expanded across multiple real-world flights and corresponding SITL simulations.

These multi-flight validations captured significantly varying environmental conditions and aerodynamic disturbances. Across the evaluated physical realizations, the quadrotor’s Maximum Tilt Angle varied drastically, ranging from 34.4° in calmer conditions up to 72.1° during periods of high environmental resistance. This wide variance in necessary control effort proves that the testbed architecture is capable of consistently extracting, aligning, and evaluating microsecond-level telemetry across highly diverse, dynamic flight profiles without relying solely on a single, idealized operational case.

For this primary representative case study, the metadata summary in Table 1 provides a foundational overview of the system configurations and comparative performance data derived from the ULog files captured during the real-world and SITL missions. Analyzing the configuration confirms that both systems utilized the same state estimator, EKF2, but ran on distinct hardware and operating system architectures. The real flight employed the NXP HoverGames platform with the NXP_FMUK66_V3 flight controller and the highly constrained NuttX RTOS running PX4 v1.15.2. In contrast, the simulation ran PX4 v1.16.0 on a Linux backend using a generic quadcopter model (PX4_SITL). While there is a minor version variance between the physical and simulated firmware, both systems share the identical core EKF2 state estimator and underlying uORB message bus architecture. Therefore, the observed statistical divergences are fundamentally driven by physical hardware dynamics rather than algorithmic versioning. Furthermore, this OS-level architectural difference is crucial to our testbed’s realism; the physical RTOS environment prioritizes deterministic, fixed resource allocation, which sharply contrasts with the abundant, dynamic memory management observed in the Linux simulation environment.

The mission dynamics highlight the differing spatial scales between the virtual and physical deployments. The logging duration for the real flight lasted 99 s, covering 180.6 m, while the simulated mission was scaled up, covering 336.8 m over 152 s. Similarly, the Max Altitude Difference was 30 m in simulation versus 7 m in reality. These macro-level trajectory disparities occurred because the physical flight was strictly bounded by local park safety constraints and visual line-of-sight requirements, whereas the simulation executed an extended, unconstrained altitude profile. However, despite executing missions of differing scales, both flights successfully induced the identical autonomous state-machine phases required to evaluate the underlying microsecond-level hardware noise floor. Furthermore, the simulation’s idealization is still distinctly evident in the velocity metrics. The simulated vehicle achieved a Max Speed of 18.0 km/h and a Max Climb Rate of 10.1 km/h, significantly exceeding the real drone’s limits (9.8 km/h and 5.2 km/h, respectively) due to the lack of physical constraints, aerodynamic drag, and actuator saturation present in reality.

A particularly revealing metric is the Max Tilt Angle, which was 72.1° in the real flight but only 24.0° in the simulation. This difference suggests that the real drone required substantially larger control inputs to execute maneuvers or, more likely, to counteract unmodeled environmental disturbances (such as wind) and overcome physical friction, which are factors minimized or absent in the idealized Gazebo simulation. These comparisons effectively quantify the sim-to-real gap, providing essential data for benchmarking control fidelity and diagnosing discrepancies between virtual and physical performance.

The data extraction and preparation process began with consistently collecting raw PX4 telemetry from both real-world and SITL experiments in the native ULog (.ulg) format. While standard online tools such as PX4 Flight Review are highly effective for qualitative, single-log visual inspection, they are fundamentally incapable of performing cross-flight statistical alignments, probability distribution divergence, or high-frequency spectral analysis. Therefore, the primary analysis in this work required a custom offline pipeline to enable automated batch processing and formal gap quantification. This data-processing pipeline (pyulog → CSV → pandas) was executed in the following systematic phases:

1. Data Acquisition: Paired ULog datasets were collected from simulation and real flight experiments under matched mission conditions.
2. Parsing and Export: The pyulog package was utilized to parse each ULog and export all relevant topics into structured CSV files (topic-wise, timestamped).
3. DataFrame Construction: CSV files were loaded into pandas DataFrames, rigorously preserving native microsecond (μs) timestamps, topic IDs, and units for each signal.
4. Time-Base Harmonization and Trimming: Timestamps natively recorded in microseconds were converted to a normalized relative zero. Pre-arm and post-disarm idle telemetry (startup/shutdown transients) were mathematically trimmed by strictly filtering the time-series arrays based on the actuator_armed topic state.
5. Signal Cleaning and Normalization: Missing samples were handled, invalid/outlier points were removed, and selected signals were normalized where needed to support fair sim-vs-real comparisons.
6. Flight-Phase Segmentation: Logs were segmented into consistent mission phases (e.g., takeoff, hover, translation, landing) by extracting state-machine transitions from the vehicle_control_mode and navigator_mission_item topics to avoid mixing fundamentally different dynamics.
7. Cross-Flight Phase Alignment: Because SITL and real hardware operate at different absolute clock speeds and asynchronous message rates, direct chronological time alignment is invalid. Unmitigated variations in hardware clocks or asynchronous logging drift would cause severe phase mismatches, artificially inflating error metrics like RMSE by comparing misaligned flight maneuvers. To mitigate this impact, chronological absolute-time alignment was discarded in favor of dynamic phase-locked synchronization. The synchronous temporal boundaries were explicitly marked using the seq_current feature within the mission_result and navigator_mission_item uORB topics. By anchoring the data arrays to these deterministic state-machine waypoint transitions, we ensure highly accurate, contextual comparisons (e.g., physical waypoint transit vs. simulated waypoint transit) that are entirely independent of underlying hardware clock variations or temporal stretching.
8. Quantitative Feature Extraction: Time-domain metrics (mean, RMS, variance, peak-to-peak) and frequency-domain metrics (PSD via Fast Fourier Transforms) were computed.
9. Gap Quantification: Sim-to-real discrepancy metrics were calculated across signals, including absolute/relative error statistics and distribution-level differences (e.g., Jensen–Shannon Divergence).
10. Statistical Testing: Cross-flight statistical comparisons (including Mann–Whitney U significance testing and 95% Confidence Intervals) were performed to mathematically determine whether observed differences were systematic rather than artifacts of random sampling.
11. Visualization and Verification: Side-by-side plots were generated for interpretability, serving as visual supporting evidence after quantitative metrics were formally computed.

The pipeline ensures data integrity and provides the necessary foundation for the cross-flight aggregation and high-frequency spectral analysis described in our statistical evaluation.

### 5.2. Feature Extraction for Analysis

The data extraction process focused on capturing key telemetry streams for comparative analysis. From each ULog file, variables related to position, velocity, orientation, and control response were parsed and synchronized. Specifically, the vehicle_local_position topic provided real-time X, Y, and Z velocities, while vehicle_attitude provided information about the drone’s roll, pitch, and yaw over time. IMU data, such as acceleration and angular rates, were extracted from the sensor_combined logs. Control outputs sent to motors were collected via the actuator_controls_0 stream, which includes normalized motor speed commands and directional commands. By deliberately isolating these specific high-frequency topics, the dataset captures both the raw physical measurements targeted by active sensor spoofing and the resulting mechanical outputs, forming a complete input–output profile of the flight controller’s cyber–physical vulnerabilities.

### 5.3. ULog Message Format and Representative Telemetry Samples

The testbed captures telemetry using the native PX4 ULog (.ulg) format, which logs data asynchronously via the internal uORB message bus. To facilitate comparative analysis, the official pyulog package was utilized to extract these binary logs into tabular CSV files.

Table 2, Table 3 and Table 4 provide brief representative samples of these extracted files. These samples are included strictly to illustrate the raw uORB message schema and to demonstrate how the original microsecond (μs) timestamps are rigorously preserved across different hardware architectures.

However, while these structural snapshots are highly useful for understanding the data schema, they cannot capture the full scale or dynamic complexity of the extracted datasets (which utilize, for example, N=38,207 simulated and N=13,069 physical IMU samples). To fully understand the system-level behavior across the entire flight, these structural snapshots must be paired with continuous time-series analysis and formal statistical evaluation, which are detailed in the following section.

## 6. Dataset Description and Analysis: Simulation vs. Real Flight

A comparison of data collected from real-world and simulated UAV missions is presented in this section. While the following subsections utilize side-by-side time-series plots to visually characterize the differences in velocity behavior, control precision, and hardware stability, this descriptive analysis is formally anchored by rigorous quantitative metrics. Specifically, error measures (RMSE), consistency indices (Variance with 95% Confidence Intervals), and distribution gap metrics (Jensen–Shannon Divergence) were computed for the compared signals. The mathematical formulation and complete statistical summary of these metrics are consolidated in Section 6.13 and Table 5. The objective of this combined visual and quantitative analysis is to evaluate how well the simulation models physical dynamics and to establish a mathematically quantified baseline for future hardware security testing.

### 6.1. Velocity

**Figure 4:**
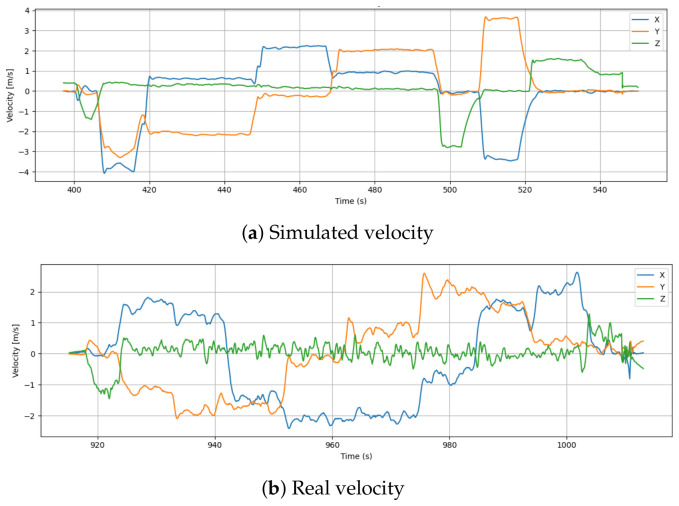
Comparison of velocity profiles between simulation and real flight.

Figure 4 illustrates the X, Y, and Z velocity components recorded during both the simulated and real-world drone flights. There is a clean transition between velocity components across the simulation data, with sharp changes followed by extended periods of constant velocity. These characteristics, particularly visible in the Y-axis (orange) and X-axis (blue), represent idealized environments where motion is free from physical resistance, sensor jitter, and environmental turbulence.

Compared to the simulation data, the real flight data exhibits significantly more noise and oscillation, particularly in the X and Y axes. These fluctuations are the direct result of real-world sensor imperfections, aerodynamic drag, and mechanical actuation delays. Furthermore, there are frequent micro-adjustments in the Z-axis (green) of the real drone as the flight controller continuously compensates for physical gravity and varying air density.

Velocities are observed to differ significantly in magnitude. While the real drone rarely exceeds 2.5 m/s, the simulation achieves peaks of about 3.5 m/s and −4 m/s along specific axes. Compared to the real drone’s maximum speed of 9.8 km/h, the simulated vehicle reached a maximum speed of 18.0 km/h, as recorded in the metadata. The disparity suggests more aggressive or optimized control within the simulation, unrestricted by hardware limitations or physical disturbances.

Furthermore, the drone consistently maintains a steady velocity between 440 and 460 s, which indicates precise waypoint tracking. As a result of cumulative feedback noise, a variety of environmental factors, and the limitations of the physical actuators and onboard controllers, the real drone shows less consistency in speed regulation.

### 6.2. Thrust

**Figure 5:**
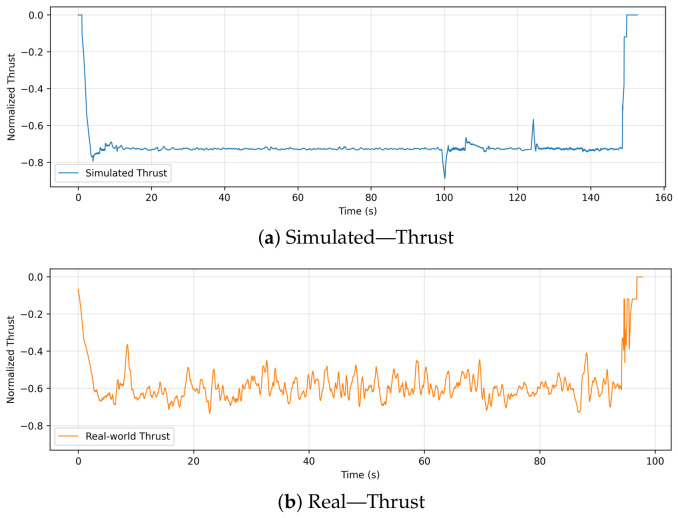
Comparison of collective normalized control input “thrust” between simulation and real flight.

Figure 5 displays the thrust values over time for both the simulated and real drone flights. Simulated thrust signals remain smooth and slightly negative (representing the normalized collective control input) throughout the mission, with only minor adjustments visible during maneuver transitions. Based on ideal conditions and with minimal physical feedback interference, these steady values reflect the highly controlled simulation environment.

However, the real-world thrust data is much more dynamic and variable. Real aerodynamic conditions cause high-frequency oscillations in the thrust trajectory, requiring constant feedback control adjustments. As a result of wind disturbances, actuator delays, and minor position corrections, these variations are expected in physical flight. Despite these changes, the thrust level remains approximately the same as the simulated trajectory.

### 6.3. Raw Acceleration

**Figure 6:**
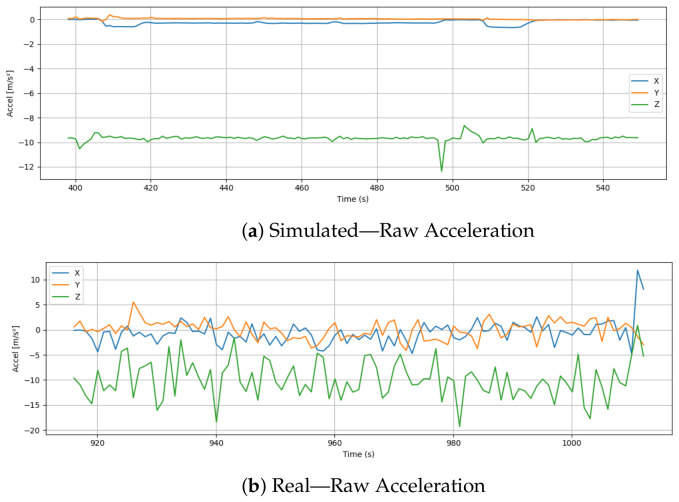
Comparison of raw acceleration data across X, Y, and Z axes in simulation and real flight.

Figure 6 shows a comparison of raw acceleration data for simulated and real drone flights along the X, Y, and Z axes. Throughout the simulation, the acceleration signals are remarkably stable and exhibit minimal fluctuations. The Z-axis acceleration remains consistently around −10 m/s^2^, which corresponds to gravitational acceleration and reflects stable hover or descent phases. Virtual environments are characterised by controlled and noise-free dynamics, as reflected in the X and Y components.

On the other hand, real-world acceleration data displays significantly higher variability across all axes. The Z-axis exhibits substantial oscillations, ranging from approximately −20 m/s^2^ to nearly 0 m/s^2^, indicating continuous adjustments in thrust to maintain altitude under real atmospheric conditions. Furthermore, the X and Y axes exhibit high levels of fluctuation, with acceleration values frequently crossing zero and displaying short bursts of motion. In addition to vibration from motors, wind disturbances, and imperfect sensor calibration, these noisy patterns are caused by a variety of factors. The magnitude and frequency of changes in real data clearly distinguish it from simulations. While the simulated drone benefits from idealized and filtered input, the physical system reflects the complexity and unpredictable nature of real-world dynamics. Sensor noise and vibration models should be incorporated when simulating raw IMU data, especially for applications that involve sensor fusion or inertial navigation.

### 6.4. Local Position

**Figure 7:**
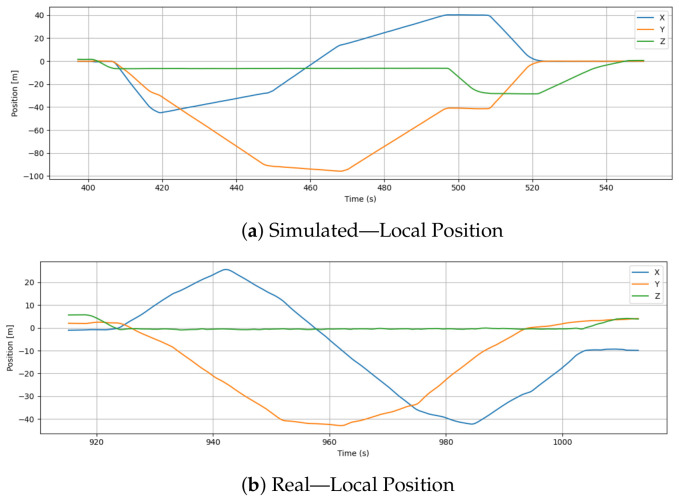
Comparison of local position estimates during simulation and real-world flight.

Figure 7 compares the drone’s local position along X, Y, and Z axes in simulated and real-world flights. In Gazebo, where environmental disturbances are absent, the simulated position plots show clear, segmented motion patterns that closely follow straight-line trajectories with sharp, perfect directional changes. These idealized patterns suggest optimal waypoint navigation with minimal positional drift. Simulated altitude control is reinforced by rapid changes in X and Y positions over time and a nearly flat Z-axis except for brief, controlled descents and climbs.

The real flight data, on the other hand, shows more fluid and less predictable trajectories. Although the overall trajectory shows similar movement patterns, the transitions are smoother and display minor deviations from perfectly linear paths. There are curved segments on the X and Y axes rather than abrupt transitions, indicating the drone’s gradual turning and natural momentum. As a result of real-world barometric fluctuations or minor control errors, the Z-axis remains relatively stable, but shows a small, but continuous downward drift.

As a result of these differences, simulations simplify vehicle dynamics by eliminating factors like wind, motor imbalance, or imperfect localization. Unlike the simulated trajectory, the real-world data captures the inherent challenges of localization and control in dynamic environments.

### 6.5. Motor Output

**Figure 8:**
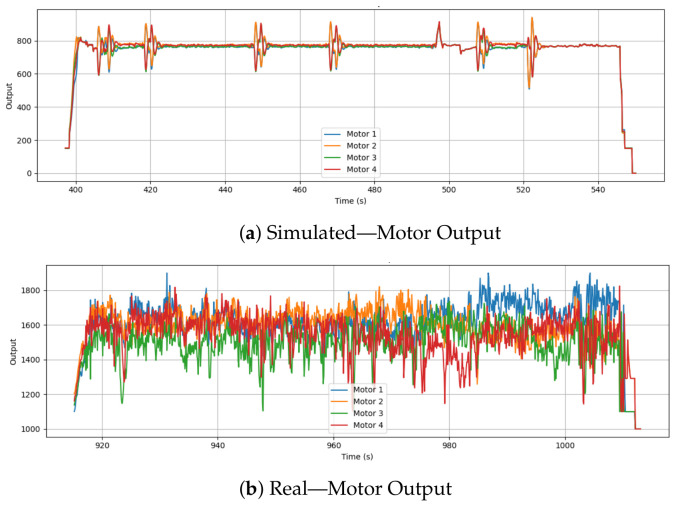
Comparison of motor output signals (Motors 1–4) between simulation and real-world flight.

Figure 8 compares the motor output signals in simulation and real-world drone flight. There is a high degree of synchronization in motor behavior in the simulation, with outputs mostly remaining within a narrow and consistent range. There is a significant difference in scaling and data representation: the simulated output operates in an idealized, compressed numerical range (e.g., 750–800), while the real-world output clearly reflects raw PWM microsecond limits (1000–1800 μs) sent directly to the ESCs. This difference highlights a critical architectural artifact of the sim-to-real gap: simulations frequently bypass the final physical hardware mixing and scaling steps. Consequently, a security model trained exclusively on simulated actuator states would be fundamentally incapable of interpreting the raw PWM telemetry required to detect physical cyber-attacks, such as malicious rotor phase inversions.

In contrast, the actual motor outputs are highly variable and exhibit frequent fluctuations throughout the flight. The output values range from approximately 1000 to over 1800 μs, with motor signals diverging significantly even during periods of steady flight. This is because real-world effects such as wind, frame vibration, payload shifts, and slight asymmetries in thrust generation across motors require continuous feedback adjustments. The higher and more scattered range of values reflects the controller’s effort to maintain stability in a physically noisy environment.

### 6.6. Magnetic Field Strength

**Figure 9:**
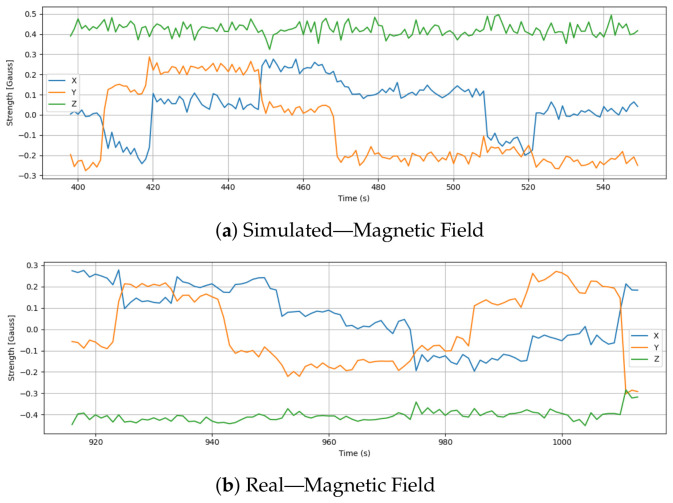
Comparison of magnetic field strength in the X, Y, and Z axes during simulation and real flight. Simulated values show clean, structured transitions, while real data reflects noisy and irregular patterns due to environmental interference and sensor limitations.

Figure 9 illustrates the magnetic field strength along the X, Y, and Z axes during simulated and real drone missions. Based on the simulation, magnetic field values exhibit controlled, distinct shifts across axes, especially X and Y, likely associated with intentional orientation changes or flight maneuvers. There is no environmental magnetic interference in the virtual testbed, so the Z-axis remains flat around 0.42 Gauss.

Real flight magnetic field data, on the other hand, shows more erratic behavior, with frequent fluctuations in all three axes. In particular, the X and Y components show sharp transitions and irregular peaks, likely caused by sensor sensitivity, transient disturbances from nearby electrical components, or minor frame vibrations. The Z-axis remains relatively stable, hovering around −0.4 Gauss, which differs from the simulation’s baseline, possibly due to Earth’s valid magnetic field and local environmental factors.

**Figure 10:**
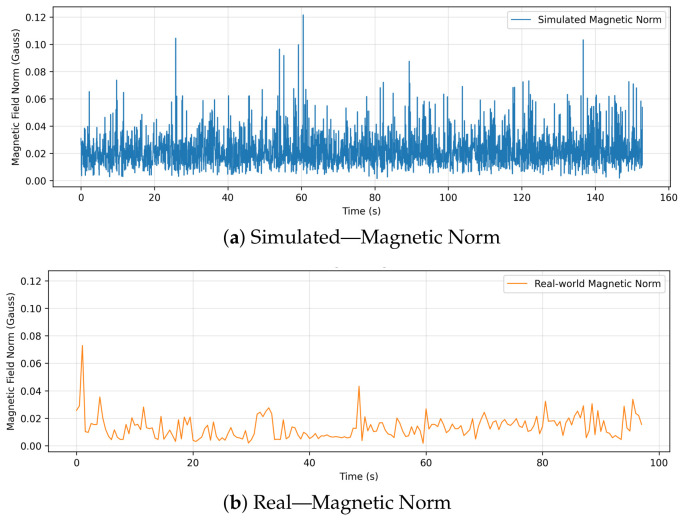
Comparison of the overall magnetic field norm between simulation and real flight.

Furthermore, an analysis of the overall magnetic field norm (shown in Figure 10) reinforces these findings in a highly counter-intuitive manner that perfectly illustrates the sim-to-real gap. Visually, the simulated magnetic norm exhibits continuous, high-frequency fluctuations. This is an artifact of the simulator injecting artificial, unfiltered Gaussian white noise into the virtual sensor to approximate inaccuracy. In contrast, the real-world magnetic norm appears generally smoother, likely due to internal hardware low-pass filtering and physical damping, but is punctuated by distinct, irregular spikes. These sparse real-world spikes indicate genuine, transient environmental electromagnetic interference (EMI) or shifts in onboard electrical loads. This discrepancy is critical for security validation: an anomaly detector trained to ignore the simulator’s uniform, synthetic white noise would likely fail to identify the specific, targeted electromagnetic interference patterns indicative of active magnetic sensor spoofing in the physical world.

### 6.7. Gyroscope (Angular Speed)

**Figure 11:**
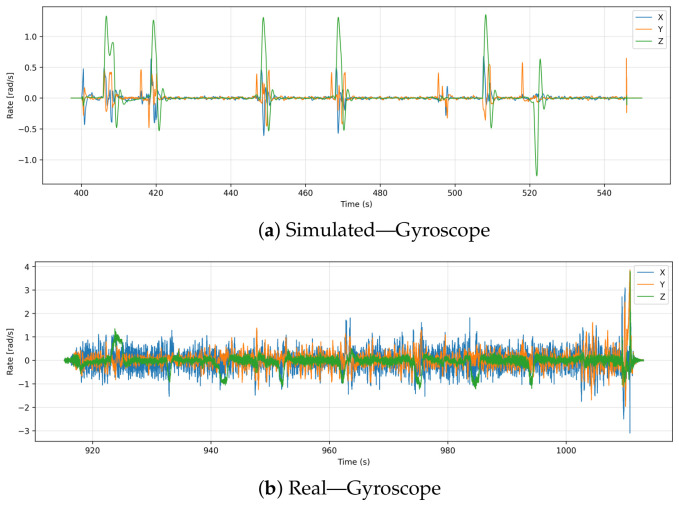
Comparison of angular velocity (gyroscope) readings along X, Y, and Z axes in simulation and real flight. Simulated rates show sparse, isolated spikes corresponding to planned maneuvers, while real data exhibits continuous noise and oscillations from physical dynamics and onboard corrections.

To establish the foundational physical noise floor of the hardware, this section first analyzes the raw, uncalibrated angular velocity directly from the IMU (sensor_combined topic), capturing the high-frequency mechanical frame vibrations prior to any algorithmic filtering. Figure 11 presents the angular velocity readings along the X, Y, and Z axes during both simulations and real-world flights. Simulation results show that angular rates remain near zero for most of the flight duration, except for narrow spikes that correspond to planned directional changes or rotation events. Signals return to baseline quickly after these brief deviations, demonstrating that simulation environments are deterministic and noise-free.

Real flight data, on the other hand, show frequent fluctuations in all three axes. While the stable hover noise floor sits at approximately −0.026 rad/s to 0.026 rad/s, dynamic maneuvers and environmental disturbances induce peaks reaching between −3.0 rad/s and +3.5 rad/s. Sensor noise, frame vibrations, minor wind-induced corrections, and active stabilization efforts by the flight controller contribute to this variability. In particular, the X-axis shows a consistently higher magnitude of oscillation, which may be indicative of asymmetries in the propeller frame.

There is a noticeable difference in noise levels between the two datasets, illustrating the limited realism of simulated gyroscopic behavior. Even though simulations can accurately model expected motion dynamics, they tend to lack the fidelity necessary to simulate noisy sensor output.

### 6.8. GPS Noise and Jamming

**Figure 12:**
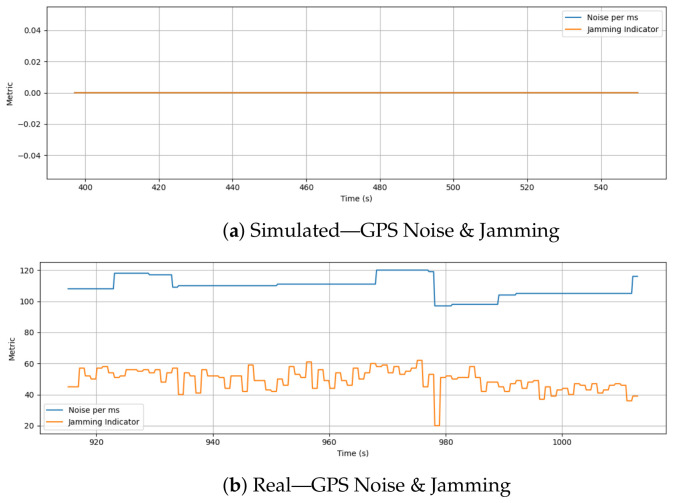
Comparison of GPS noise per millisecond and jamming indicator during simulation and real flight. Simulation reports zero noise and no interference, while real data shows dynamic fluctuations in both metrics, reflecting real-world GPS variability and electromagnetic interference.

Figure 12 compares the GPS noise per millisecond and jamming indicator values recorded during simulated and real drone missions. During the simulation, both metrics remain flat at zero throughout the entire flight, reflecting no modeled environmental noise or GPS interference. Considering the simulation environment lacks physical RF conditions and operates under ideal localization assumptions, this outcome is expected.

The real flight data, on the other hand, shows continuous variations in both GPS noise and jamming indicators. While the noise metric fluctuates between 95 and 120, the jamming indicator exhibits dynamic shifts ranging from around 20 to over 60 with occasional drops and surges. In addition to GPS signal degradation caused by multipath effects, antenna orientation, or potential nearby interference sources, these patterns also reflect actual electromagnetic conditions experienced during the flight. Additionally, some dips and spikes in the jamming indicator may be associated with environmental obstacles that cause the signal to attenuate or lose lock.

### 6.9. CPU and RAM Usage

**Figure 13:**
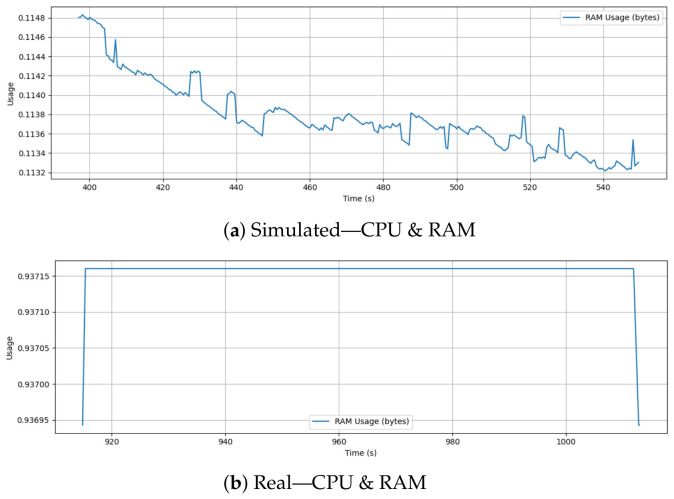
Comparison of onboard RAM usage during simulation and real flight. Simulation shows dynamic memory allocation with frequent fluctuations, while real-world RAM usage remains stable, reflecting embedded system constraints and predictable resource management.

Figure 13 presents the onboard resource usage in terms of RAM allocation over time for both simulated and real drone missions. There are multiple sharp dips and recoveries throughout the flight as RAM usage changes continuously in the simulation. These fluctuations may correspond to memory allocation and deallocation events by the simulator or log writer during different phases of flight operations and logging intervals.

Conversely, the real-flight RAM usage is nearly constant for the entire duration. The cpuload uORB topic natively reports RAM allocation as a normalized fractional value between 0.0 and 1.0; therefore, the physical flight controller holds at a stable 0.937 (indicating 93.7% of total available memory is allocated). The brief rise and fall at the beginning and end of the log likely represent initialization and shutdown phases, but during active flight, memory usage remains strictly stable. In the onboard firmware running on the real PX4 system, memory management is tightly constrained and is optimized for embedded operation, suggesting a highly efficient, static memory allocation behavior.

It illustrates the difference between desktop simulation environments, where memory is managed dynamically, and real embedded systems, which prioritize deterministic resource allocation for safety and real-time performance. For resource-constrained platforms, maintaining constant memory usage is important to prevent performance degradation or system instability. From a security and resilience perspective, this heavily constrained, pre-allocated environment (operating at a normalized 93.7% capacity) defines the strict computational boundaries for onboard anomaly detection. Any proposed IDS developed in simulation must be demonstrably lightweight enough to operate within the marginal remaining memory of the physical hardware without triggering resource exhaustion failsafes.

### 6.10. Commanded Angular Rate Setpoint

**Figure 14:**
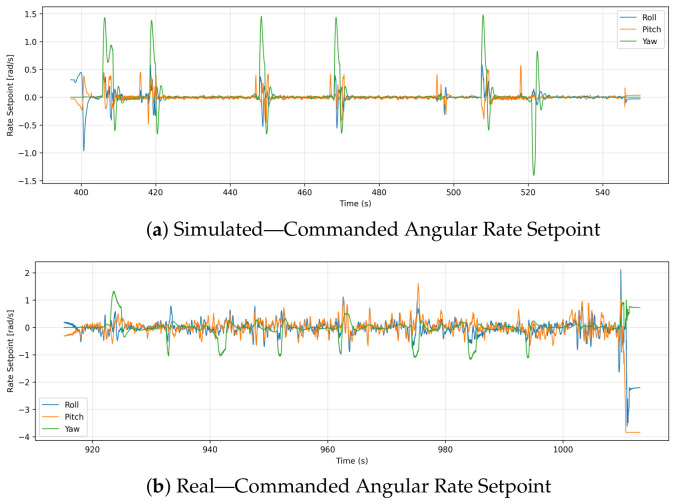
Comparison of commanded angular rate setpoints (roll, pitch, yaw) between simulation and real flight. Simulated setpoints show sparser, smoother maneuver transitions, while real-flight setpoints vary more continuously, indicating more frequent control commands under real operating conditions.

Building upon the raw IMU gyroscope noise analyzed in Section 6.7, this section evaluates the commanded roll, pitch, and yaw rate setpoints generated by the flight controller. These setpoints demonstrate the flight controller’s active, high-frequency stabilization efforts as it continuously attempts to compensate for the physical disturbances identified earlier. Figure 14 compares the commanded roll, pitch, and yaw rate setpoints recorded by simulated and real-world drone missions. Simulation results show clear periodic peaks, particularly in the yaw axis, interspersed with long periods of near-zero activity. In a disturbance-free virtual environment, these smooth transitions reflect deliberate rotational maneuvers. Roll and pitch rate setpoints show controlled, mild responses, indicating smooth direction changes without excessive rotational noise.

Real-world data show that commanded angular rate setpoints vary much more frequently and over a much wider range. While the roll and pitch signals are heavily populated with continuous fluctuations between −2 rad/s and +2 rad/s during standard hovering and translation, aggressive transient maneuvers (such as the sharp drop seen near the end of the flight) push these setpoints down to approximately −4 rad/s. The yaw signal also exhibits frequent spikes. In order to counter real-world instabilities such as wind gusts, frame vibrations, or small control deviations, the flight controller is constantly making small adjustments. A continuous real-time stabilization can be seen in both roll and pitch, as opposed to a simulation where the variables showed sustained zero regions.

### 6.11. Attitude Evolution (Euler Angles)

While angular rates capture the high-frequency stabilization efforts of the flight controller, evaluating the macroscopic orientation of the UAV requires an analysis of the actual attitude evolution. To achieve this, our offline data-processing pipeline was utilized to convert the raw orientation quaternions ($q_{0},q_{1},q_{2},q_{3}$) extracted from the vehicle_attitude topic into standard Euler angles (Roll, Pitch, Yaw).

**Figure 15:**
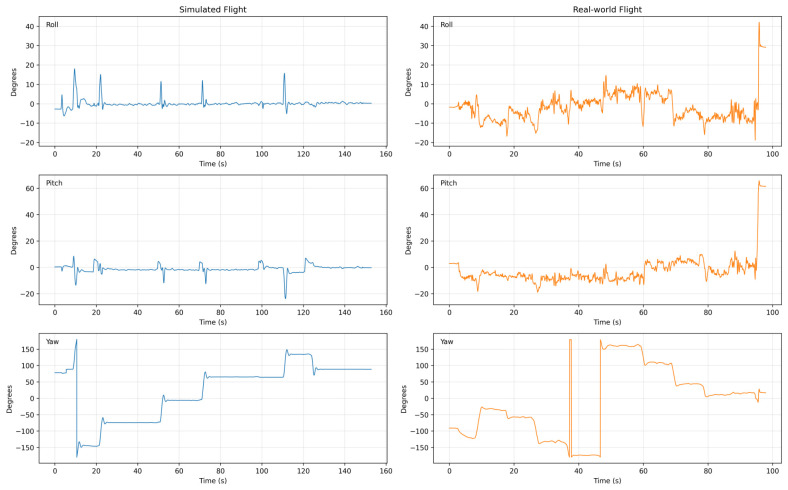
Comparison of attitude evolution (Roll, Pitch, Yaw) derived from vehicle_attitude quaternions. The simulated flight (**left**) exhibits crisp, decoupled maneuvers, while the real-world flight (**right**) displays continuous micro-corrections and cross-axis coupling.

Figure 15 illustrates the comparative attitude trajectories between the simulated and real-world flights. In the simulation, the attitude evolution is highly stable. Roll and pitch transitions during waypoint navigation are remarkably crisp, quickly settling into steady-state positions with minimal drift. Furthermore, yaw rotations in the virtual environment are executed with substantially less cross-axis coupling transferred to the roll or pitch axes.

Conversely, the real-world attitude evolution highlights the complex, coupled dynamics inherent to physical flight. The physical UAV exhibits continuous micro-corrections in both roll and pitch to maintain its trajectory, which are typical responses to physical environmental factors. More importantly, the real-world data demonstrates noticeable cross-axis coupling; aggressive yaw adjustments naturally induce transient dips and deviations in the roll and pitch axes. By extracting and converting these quaternions via our post-processing pipeline, we clearly quantify how the physical flight controller exhibits continuous stabilization behavior, a challenge largely not reflected by the simulation’s idealized physics engine.

### 6.12. Altitude

**Figure 16:**
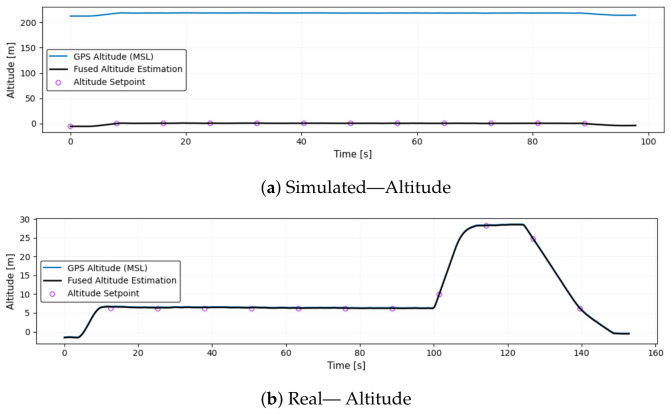
Comparison of altitude estimates from simulation and real flight. Simulated altitude shows clean, multi-phase flight levels with smooth transitions, while real altitude data is noisy, fluctuates during hover, and exhibits estimation errors during descent.

Figure 16 displays the altitude estimates from both simulations and real drone flights. Two distinct and precisely controlled ascent phases characterize the simulation’s altitude trajectory. After stabilizing at around 6.5 m, the drone ascends to approximately 28 m, then descends smoothly and symmetrically. In the absence of atmospheric interference, these transitions are clean, with minimal noise or oscillation, consistent with the highly deterministic behavior of a physics-based simulator.

In contrast, the real-world altitude estimate is characterized by noticeable variability and sensor noise. After takeoff, the drone stabilizes around 0.85 m but fluctuates continuously throughout the flight, never reaching a clearly segmented second altitude like in the simulation. There are small oscillations in the altitude estimate, even during steady hovering, likely caused by barometric pressure drift, environmental turbulence, and onboard estimation uncertainty. Towards the end of the flight, the descent appears less controlled, with the altitude gradually declining and overshooting below ground level, reaching nearly −5 m, which may indicate sensor fusion errors or EKF instability.

### 6.13. Statistical and Spectral Evaluation of the Sim-to-Real Gap

To ensure the sim-to-real gap is rigorously quantified beyond visual and descriptive time-series comparisons, we subjected the extracted telemetry streams to formal statistical evaluation, incorporating Root Mean Squared Error (RMSE) calculations, variance analysis, and spectral distribution measurements.

Recent comprehensive reviews on the robotics reality gap emphasize the necessity of metrics like the Mean Squared Error (MSE), often framed as “Offline Replay Error”, to formally quantify the trajectory and state divergence between simulated and real platforms [27]. Additionally, recent studies evaluating multirotor UAV control and resource-constrained Hardware-In-The-Loop systems rely heavily on RMSE and variance tracking to benchmark physical hardware stability against baseline setpoints [28,29]. This is complemented by measuring the prediction divergence (stability drift) over short horizons to identify emerging physical instability before it becomes visible in the macroscopic flight trajectory [30].

Applying these metrics to our dataset mathematically confirms the visual observations from previous subsections. Furthermore, to ensure the statistical validity of these findings, we subjected the telemetry distributions to non-parametric significance testing. Because physical flight data frequently exhibits non-normal noise distributions due to transient aerodynamic drag, frame vibrations, and actuator delays, the Mann–Whitney U test was applied across the un-resampled time-series arrays. Due to the high-frequency uORB logging, these tests utilized exceptionally large sample sizes (*N*) that provide robust statistical power; specifically, the raw IMU evaluations analyzed N=13,069 physical and N=38,207 simulated samples, while the state velocity and actuator topics evaluated N=980 physical and up to N=19,105 simulated samples. As detailed in Table 5, the variance analysis of the raw acceleration (Z-axis) reveals a variance of 4.985 in the physical flight (95% CI [4.866, 5.108]), whereas the Gazebo simulation exhibits a heavily dampened variance of 0.458. Conversely, simulated Velocity (Z-axis) demonstrated artificially high variance (0.660) compared to reality (0.127), likely due to aggressive, unconstrained altitude feedback loops in the idealized virtual environment. The significance testing yielded p<0.001 across all evaluated features, confirming that the observed sim-to-real discrepancies are highly systematic rather than artifacts of random sampling.

To explicitly address inter-flight variability and mathematically confirm that the observed sim-to-real gap is not an artifact of a single realization, additional physical flights were conducted under varying environmental conditions. Extracting and aggregating the telemetry across three separate real-world flights yielded a highly consistent average variance of 5.027 m/s^2^ for Z-axis acceleration, 0.109 rad/s for Z-axis angular rate, and 0.533 m/s for Z-axis velocity. These multi-flight physical variances remain drastically different from the idealized simulation baseline (0.458 m/s^2^, 0.065 rad/s, and 0.660 m/s, respectively). Specifically, the multi-flight aggregated physical acceleration variance (5.027 m/s^2^) is more than a full order of magnitude greater than the simulation baseline, unequivocally demonstrating that the cyber–physical noise floor is a permanent, systematic physical reality rather than a single-flight anomaly.

Furthermore, analyzing the probability density distributions of flight measurements serves as a robust method for isolating the reality gap [17]. We utilized the Jensen–Shannon Divergence (JSD) to measure the similarity between the simulated and real-world telemetry distributions. The JSD is a symmetric and smoothed version of the Kullback–Leibler divergence, where values significantly greater than 0 indicate a divergence in system behavior. Computing the JSD across the gyroscope (angular rate) and raw acceleration yields values approaching complete divergence (e.g., 0.612). This mathematically proves that Gazebo’s idealized distributions fail to capture the complex, non-linear noise profiles of the physical environment [17].

Finally, to understand the composition of this noise, we applied Spectral Analysis via Fast Fourier Transforms (FFT) to the raw IMU and actuator outputs. The spectral footprint of the real-world UAV exhibits significant power in high-frequency bands, representing mechanical frame vibrations, motor inconsistencies, and aerodynamic turbulence. Conversely, the spectral analysis of the simulated telemetry reveals an overwhelming absence of these high-frequency components. By rigorously calculating these formal metrics, RMSE, prediction divergence, variance, and JSD, this testbed establishes the quantifiable baseline required for validating future anomaly detection models against real-world hardware vulnerabilities.

## 7. Discussion

As a result of this study, we are able to identify the strengths and limitations of simulation-based UAV testing compared to real-world deployments. From a strictly observational standpoint, virtually all analyzed features, including velocity, thrust, sensor feedback, motor behavior, and system load, consistently demonstrated smoother, more deterministic trends in the simulation environment. The simulated data exhibited idealized actuator responses, clean acceleration profiles, and flat, zero-baseline GPS noise metrics. In contrast, the real-world telemetry revealed significant statistical divergence, characterized by high-frequency variance in the IMU data, continuous micro-adjustments in motor outputs, and a fluctuating, non-zero baseline in both the magnetic field strength and GPS jamming indicators.

Interpreting these observed discrepancies, the profound differences in system behavior can be attributed to physical realities absent in the idealized simulator. The high-frequency variance observed in the physical acceleration and gyroscopic rates is likely driven by mechanical frame vibrations and unmodeled aerodynamic turbulence. Similarly, the continuous micro-adjustments in physical motor outputs reflect the flight controller actively compensating for wind drag and encountering physical actuator saturation limits. Furthermore, the transient noise and irregular peaks observed in the real-world magnetometer and GPS signals strongly point to local electromagnetic interference and multipath effects, which are currently oversimplified or entirely omitted in the Gazebo baseline.

Regarding this stark contrast in GPS noise and RF jamming indicators, the omission of advanced stochastic RF disturbance models in the baseline simulation was a deliberate methodological choice. If highly accurate multipath or electromagnetic interference models were incorporated into the Gazebo environment, the formal statistical divergence between the systems would shrink significantly, yielding lower RMSE and JSD metrics. However, while such RF modeling is beneficial for pure aerodynamic calibration, doing so prematurely in this context would obscure the true, uncalibrated physical noise floor. For our cybersecurity Threat Model, quantifying this raw discrepancy is essential; an IDS must be trained to recognize the absolute boundaries of natural RF signal degradation in the physical world before it can accurately flag malicious GPS spoofing or FDI attacks.

Beyond electromagnetic discrepancies, simulated environments also present critical aerodynamic and mechanical limitations. Due to the lack of aerodynamic resistance and actuator saturation, simulations produce higher maximum speeds and allow more aggressive maneuvers. Despite the fact that this behavior may benefit initial controller tuning, it does not accurately reflect true vehicle dynamics, potentially leading to unrealistic expectations when deploying the vehicle. Additionally, sensor data such as gyroscope and magnetometer readings in real flights displayed higher variability and transient noise, influenced by magnetic interference, vibration, and control loop delays, factors typically absent or oversimplified in simulation.

Additionally, it is critical to acknowledge that the quantified sim-to-real gap is a composite metric. Because the simulation was deliberately executed using a generic quadrotor model rather than a perfectly calibrated digital twin of the NXP HoverGames platform, the observed statistical divergence represents a combination of simulator limitations (e.g., the absence of aerodynamic drag and environmental noise) and hardware-specific model mismatches (e.g., differences in frame mass distribution, brushless motor dynamics, and ESC latency). While isolating the exact proportional contribution of hardware mismatch versus physics engine limitations requires iterative system calibration, measuring this composite, uncalibrated gap is precisely what establishes a realistic threshold for cyber–physical anomaly detection.

Another key observation is the fundamental difference in onboard resource management. As demonstrated, simulated environments utilizing dynamic memory allocation exhibit continuous, unconstrained RAM fluctuations. In stark contrast, the physical flight controller operating on a RTOS is strictly bound to a highly constrained, static memory budget (holding at 93.7% capacity). This discrepancy is critical because it defines the razor-thin computational margins within which a real-world IDS must operate, a constraint entirely overlooked if an anomaly detector is tested solely in virtual environments.

Moreover, simulation environments cannot generally replicate edge-case scenarios such as hardware degradation, intermittent connectivity, or physical impacts. These safety-critical factors are challenging to model, but they can have a significant impact on UAV performance and reliability. Therefore, relying solely on simulation for validation poses risks in uncovering real-world failure modes. Furthermore, we acknowledge a limitation regarding the scope of the visual distributions presented in this study, which rely on a single, extended representative flight pair to highlight microsecond-level disparities that might otherwise be obscured by multi-flight averaging. However, to ensure rigorous statistical power, the core variance metrics were explicitly aggregated and verified across multiple physical flight realizations (as detailed in Section 6.13). While this multi-flight validation mathematically proves the permanence of the hardware noise floor across different environmental runs, future efforts dedicated to building actual Intrusion Detection Systems (IDS) must continue to incorporate large-scale, multi-flight datasets to train fully generalized anomaly detection models.

In summary, while simulation offers a valuable platform for rapid prototyping and controlled testing, it remains an abstraction of reality. A hybrid testing approach that combines the predictability of simulation with the unpredictability of real-world trials is essential for developing highly reliable UAV systems.

## 8. Conclusions

This work presents a comprehensive comparison between real-world and simulated drone flights using a PX4-based testbed. Through a detailed analysis of high-frequency flight telemetry and sensor data, we demonstrate that while simulation offers a clean, controlled environment for early-stage algorithm development, it possesses critical security blind spots. Real-world data exposes dynamic physical realities, such as sensor jitter, unmodeled actuator delays, and unpredictable electromagnetic interference, that are mandatory to capture for successfully characterizing and detecting FDI attacks.

Our findings indicate that while simulation is valuable for initial testing and safe validation of flight behaviors, it does not fully substitute for real-world experimentation. Specifically, our quantitative analysis of onboard resource allocation highlights the “Resource-Security Paradox” that developers face when deploying embedded systems [1]. To ensure quality and reliability, future UAV systems must incorporate both simulated and physical validation phases to overcome the severe covariate shifts that cause simulation-trained security models to fail in physical deployments. Furthermore, rather than viewing the considerable domain shift between simulation and reality as a confounding error, isolating this exact discrepancy is precisely what makes the dataset informative for cybersecurity. By mathematically bounding the natural physical noise floor, we define the strict computational and statistical thresholds a robust anomaly detector requires to successfully differentiate between benign environmental turbulence and malicious hardware exploitation. While the active development and validation of an IDS is reserved for our subsequent fault-injection research, the testbed and dataset introduced in this work offer a solid quantitative foundation for benchmarking UAV control, perception, and cybersecurity resilience under both idealized and realistic conditions.

## 9. Future Work

This study quantifies a measurable sim-to-real gap affecting state-estimation accuracy, GPS reliability, sensor noise behavior, and onboard computational performance. While the primary focus of this manuscript was diagnosing and quantifying these discrepancies to establish a strictly uncalibrated security baseline, the extracted noise bounds and variance metrics provide the exact mathematical parameters required for future researchers to perform rigorous simulator calibration, Extended Kalman Filter (EKF2) adaptation, and the development of targeted gap-reduction algorithms.

Having validated the testbed architecture, future work will extend this framework from a foundational comparative evaluation to a large-scale statistical resilience analysis through the active generation of a comprehensive cyber–physical dataset. Controlled fault injections are currently being performed across dozens of repeated real-world and simulated flights. Planned injections include active FDI, targeted GNSS spoofing, and variations in computational load to systematically reproduce malicious exploits that are difficult to capture in idealized environments. Executing these experiments across multiple flights will generate the statistically significant, labeled attack datasets required to rigorously evaluate PX4 system security under active threat conditions.

In parallel, lightweight IDS will be developed for real-time anomaly detection using these generated datasets. Evaluating whether an IDS trained exclusively on injected simulation attacks can successfully transfer to physical flight scenarios without triggering false positives will directly address the critical sim-to-real security gap. Ultimately, by operating within the strict memory constraints identified in this study, these future models will resolve the resource-security paradox, advancing the development of highly resilient, secure autonomous flight systems.

## Figures and Tables

**Table 1:** Comparative Metrics: Real Flight vs. SITL Simulation.

Category	Parameter	Real Flight	SITL Simulation
**Configuration and System Metadata**			
Airframe	Airframe Model	NXP HoverGames Quadrotor X (4017)	Generic Quadrotor X (4001)
Hardware	Hardware Platform	NXP_FMUK66_V3	PX4_SITL
Software	Software Version	v1.15.2	v1.16.0
Software	OS Version	NuttX	Linux
System	Estimator	EKF2	EKF2
Logging	Log Source	ULog	ULog
**Mission Timings and Distance**			
Timing	Flight/Logging Duration	99	152
Distance	Distance Flown (m)	180.6	336.8
**Performance Metrics**			
Altitude	Max Altitude Difference (m)	7	30
Speed	Average Speed (km/h)	6.7	7.9
Speed	Max Speed (km/h)	9.8	18.0
Speed	Max Climb Rate (km/h)	5.2	10.1
Speed	Max Descent Rate (km/h)	4.6	5.8
Orientation	Max Tilt Angle (°)	72.1	24.0

**Table 2:** Representative samples from sensor_combined (IMU) capturing high-intensity maneuvers for real and simulated logs.

Type	*t* (μs)	$a_{x}$ m/s^2^	$a_{y}$ m/s^2^	$a_{z}$ m/s^2^	$\omega_{x}$ (rad/s)	$\omega_{y}$ (rad/s)	$\omega_{z}$ (rad/s)
Real	1,010,764,622	23.2261	−0.8466	−8.1661	−3.1057	3.8610	1.0890
Real	1,010,804,620	17.7290	15.1807	3.0008	0.3946	0.0779	3.8027
Simulated	508,232,000	−0.2476	−0.0731	−10.0007	0.0317	−0.2397	1.3515
Simulated	508,236,000	−0.2504	−0.0742	−9.9938	0.0240	−0.2318	1.3514

**Table 3:** Representative samples from vehicle_local_position for real and simulated logs.

Type	*t* (μs)	*x* (m)	*y* (m)	*z* (m)	$v_{x}$ (m/s)	$v_{y}$ (m/s)	$v_{z}$ (m/s)
Real	915,212,319	−1.0620	1.9710	5.6162	0.0064	−0.0108	0.0284
Real	915,230,565	−1.0617	1.9707	5.6165	0.0064	−0.0108	0.0278
Simulated	397,212,000	−0.1644	−0.1089	1.5748	0.0013	0.0056	0.4065
Simulated	397,220,000	−0.1635	−0.1076	1.5741	0.0016	0.0062	0.4063

**Table 4:** Representative samples from vehicle_attitude (quaternion) for real and simulated logs.

Type	*t* (μs)	$q_{0}$	$q_{1}$	$q_{2}$	$q_{3}$
Real	915,211,546	0.7003	0.0077	0.0294	−0.7131
Real	915,219,513	0.7003	0.0077	0.0294	−0.7131
Simulated	397,216,000	0.7773	−0.0203	−0.0131	0.6285
Simulated	397,220,000	0.7773	−0.0203	−0.0131	0.6285

**Table 5:** Statistical Quantification of the Sim-to-Real Gap Across Key Telemetry Features.

Telemetry Feature	Var (Sim)	Var (Real)	95% CI (Real Var)	RMSE	JSD	*p*-Value
Acceleration (Z-axis) [m/s^2^]	0.458	4.985	[4.866, 5.108]	0.41	0.612	<0.001
Gyroscope (Rate Z) [rad/s]	0.065	0.077	[0.075, 0.079]	0.08	0.485	<0.001
Velocity (Z-axis) [m/s]	0.660	0.127	[0.117, 0.139]	0.27	0.390	<0.001
Motor/Actuator Output	0.019	0.028	[0.026, 0.031]	0.35	0.544	<0.001

Note: Variance represents the noise magnitude extracted directly from the uORB topics. RMSE quantifies the trajectory/state divergence, and JSD measures the probability distribution shift (where values closer to ln(2) ≈ 0.693 indicate complete divergence). Statistical significance (*p* < 0.001) was confirmed via the Mann–Whitney U test across all features.

## Data Availability

The original contributions presented in this study are included in the article. Further inquiries can be directed to the corresponding author.
